# Chemotherapy-Induced Nausea and Vomiting

**DOI:** 10.1155/2015/457326

**Published:** 2015-09-06

**Authors:** Bernardo Leon Rapoport, Alexander Molasiotis, Haralambos Raftopoulos, Fausto Roila

**Affiliations:** ^1^The Medical Oncology Center of Rosebank, Saxonwold, Johannesburg 2196, South Africa; ^2^School of Nursing, The Hong Kong Polytechnic University, Hong Kong; ^3^Merck & Co., Rahway, NJ 07065, USA; ^4^Medical Oncology, Santa Maria Hospital, Terni, Italy

In the past two decades, significant advances have been made in the management of chemotherapy-induced nausea and vomiting (CINV). These advances are primarily due to a greater understanding of the physiological and molecular pathways underlying CINV, which resulted in major progress in the management of patients with CINV.

In the early 1990s, CINV treatment consisted of dexamethasone [1]. Improvements in the management of CINV control were achieved with the discovery of 5-hydroxytryptamine (5HT_3_) receptor and the development of 5HT_3_ receptor antagonists (RA). This pathway is primarily involved in the acute phase of CINV. Subsequent studies demonstrated that the usage of the combination of 5HT_3_ RA and dexamethasone resulted in additional improvements in CINV control [2, 3].

Over the last decade, the discovery of the neurokinin-1 receptor antagonists (NK_1_-RA) and its role in the pathogenesis of delayed phase of CINV has led to significant developments in the management of this complication of anticancer treatment. More importantly, these milestone achievements are significant and resulted in an improvement in anticancer treatment compliance, as well as an improvement in the quality of life of patients diagnosed with cancer.

Despite these achievements, nausea, in particular, and vomiting remain a clinically significant problem for patients receiving both highly emetogenic chemotherapy (HEC) and moderately emetogenic chemotherapy (MEC). Seventy percent of patients treated with cisplatin-based HEC will achieve an overall antiemetic complete response when managed with a triple therapy consisting of a NK_1_ RA aprepitant in combination with a 5HT_3_ RA and corticosteroids prophylaxis [4, 5]. The current antiemetic guidelines (MASCC/ESMO, ASCO, and NCCN) endorse triple therapy treatment for patients receiving cisplatin- and AC-based chemotherapy regimes [6–8].

The current special issue includes several reviews including the biology and pharmacology of the NK_1_ receptor and substance P, the antiemetic management of germ cell tumor patients undergoing multiple days' chemotherapy treatment, radiotherapy induced nausea and vomiting (RINV), CINV induced by oral cytotoxic agents and targeted therapies in patients undergoing treatment for solid tumors, adherence to CINV guidelines and the benefits of NEPA (a new agent consisting of a combination of netupitant and palonosetron), and the treatment of breakthrough and refractory chemotherapy-induced nausea and vomiting. Additionally, 2 original clinical papers are presented investigating the ramosetron and olanzapine in the management of CINV.

The review article by S. Garcia and P. Gascon provides an extensive overview of the basic knowledge of the NK_1_ receptor and substance P biology and the pharmacological basis of the usage of NK_1_ receptor antagonists in the management of delayed phase of CINV.

The 5-HT_3_ receptor antagonists play an important role in the pathogenesis of the acute phase of CINV. K.-R. Kim et al. presented a pilot study with the potential usage of ramosetron, a tetrahydrobenzimidazole derivative structurally independent of the previously developed 5-HT_3_ receptor antagonists, such as ondansetron, granisetron, and tropisetron.

Patients with germ cell tumor undergoing 5 days of cisplatin-based chemotherapy have a different mechanism and pattern of CINV compared to those receiving single day chemotherapy. The efficacy of antiemetic drugs, as observed in single day chemotherapy, is therefore not applicable. In this issue, P. Ranganath et al. from Indiana University discuss current recommendations and future directions for patients undergoing multiple day treatment.

Depending on the site of irradiation, dosing, fractionation, irradiated volume, and radiotherapy techniques, the incidence of nausea and vomiting after radiotherapy is approximately 50–80%. RINV is a very important and not well researched area often underestimated by physicians. K. Jordan et al. include an overview of RINV and current guidelines recommendations as well as future directions.

The treatment of nausea and vomiting caused by oral antineoplastic agents is primarily empirical, consisting of the administration of daily oral antiemetic therapy. The level of evidence of prophylactic antiemetics recommended for these agents is low. A. L. Costa et al. discuss the management of CINV induced by oral cytotoxic agents and targeted therapies in patients undergoing treatment for solid tumors. This article highlights the differences in the classification of emetogenic potential of oral antineoplastic agents between the different international guidelines, as well as different recommendations for prophylactic antiemetic treatments.

NEPA is a new oral single fixed combination agent, containing a highly selective NK_1_ RA, netupitant with palonosetron. This agent is a pharmacologically and clinically distinct 5-HT_3_ RA. Palonosetron has a longer half-life compared with older 5-HT_3_ RAs. Palonosetron works synergistically with netupitant and has the potential to improve the efficacy in the prevention of the delayed phase of CINV when used in combination. P. J. Hesketh et al. discuss the use of NEPA in the context of how NEPA may overcome some of the barriers interfering with adherence to the various antiemetic guidelines.

Several studies have shown that the antipsychotic agent olanzapine is effective in the management of CINV. The pharmacological mechanism of action consists of the blocking of neurotransmitter receptors including dopaminergic at D_1_, D_2_, D_3_, and D_4_ brain receptors, serotonergic at 5-HT_2a_, 5-HT_2c_, 5-HT_3_, and 5-HT_6_ receptors, catecholamines at alpha_1_ adrenergic receptors, acetylcholine at muscarinic receptors, and histamine at H_1_ receptors. In this special issue, R. M. Navari discusses the treatment of breakthrough and refractory chemotherapy-induced nausea and vomiting with special reference to olanzapine in this setting. In another paper, M. Abe et al. retrospectively analyze the role of olanzapine in 50 gynecologic cancer patients receiving cisplatin-based chemotherapy who had nausea despite the use of standard therapy.

The treatment of CINV is evolving and this special issue highlights some of the recent developments as well as some of the controversies in this important field of oncology. 

*******Bernardo**  **Leon**  **Rapoport***** *******Alexander**  **Molasiotis***** *******Haralambos***  ***Raftopoulos******* *******Fausto***  ***Roila*******

## References

[1] Aapro M. S., Alberts D. S. (1981). Dexamethasone as an antiemetic in patients treated with cisplatin. *The New England Journal of Medicine*.

[2] Falkson G., van Zyl A. J. (1989). A phase I study of a new 5HT3-receptor antagonist, BRL43694A, an agent for the prevention of chemotherapy-induced nausea and vomiting. *Cancer Chemotherapy and Pharmacology*.

[3] Smith D. B., Newlands E. S., Spruyt O. W. (1990). Ondansetron (GR38032F) plus dexamethasone: effective anti-emetic prophylaxis for patients receiving cytotoxic chemotherapy. *British Journal of Cancer*.

[4] Hesketh P. J., Grunberg S. M., Gralla R. J. (2003). The oral neurokinin-1 antagonist aprepitant for the prevention of chemotherapy-induced nausea and vomiting: a multinational, randomized, double-blind, placebo-controlled trial in patients receiving high-dose cisplatin—the aprepitant protocol 052 study group. *Journal of Clinical Oncology*.

[5] Poli-Bigelli S., Rodrigues-Pereira J., Carides A. D. (2003). Addition of the neurokinin 1 receptor antagonist aprepitant to standard antiemetic therapy improves control of chemotherapy-induced nausea and vomiting: results from a randomized, double-blind, placebo-controlled trial in Latin America. *Cancer*.

[6] National Comprehensive Cancer Network (2015). NCNN Guidelines 1.2012: antiemesis 1.2015.

[7] Roila F., Herrstedt J., Aapro M. (2010). Guideline update for MASCC and ESMO in the prevention of chemotherapy- and radiotherapy-induced nausea and vomiting: results of the Perugia consensus conference. *Annals of Oncology*.

[8] Basch E., Prestrud A. A., Hesketh P. J. (2011). Antiemetics: American Society of Clinical Oncology clinical practice guideline update. *Journal of Clinical Oncology*.

